# Literature review of the clinical features of sulfasalazine-induced drug reaction with eosinophilia and systemic symptoms/drug-induced hypersensitivity syndrome (DRESS/DIHS)

**DOI:** 10.3389/fphar.2024.1488483

**Published:** 2024-12-02

**Authors:** Ya Liu, Danxia Wang, Shiwei Wu, Xiang Liu, Can Xiao

**Affiliations:** ^1^ Department of Clinical Pharmacy, Xiangtan Central Hospital (The Affiliated Hospital of Hunan University), Xiangtan, China; ^2^ Department of Pharmacy, People’s Hospital of Ningxiang City, Hunan University of Chinese Medicine, Changsha, China

**Keywords:** sulfasalazine, drug reaction with eosinophilia and systemic symptoms (DRESS), drug-induced hypersensitivity syndrome (DIHS), HHV-6 reactivation, diagnosis

## Abstract

**Background:**

Sulfasalazine (SSZ) is commonly prescribed for the treatment of ulcerative colitis, rheumatoid arthritis, and ankylosing spondylitis. However, it can also trigger a severe drug reaction known as Drug Reaction with Eosinophilia and Systemic Symptoms (DRESS) or Drug-Induced Hypersensitivity Syndrome (DIHS). This article aims to analyze the clinical characteristics of DRESS/DIHS induced by SSZ and provide evidence for clinical diagnosis, treatment, and prevention.

**Methods:**

We gathered relevant literature on SSZ-induced DRESS/DIHS published from 1 January 2005, to 21 July 2024, by searching both English and Chinese databases.

**Results:**

Thirty-nine patients (15 males and 24 females) were included in the study, with a median age of 47 years (range: 11–82 years). Following SSZ administration, the median onset time of DRESS/DIHS was 28 days (range: 10–60 days). These patients exhibited clinical symptoms such as fever (100%), rash (100%), digestive system responses (38.5%), and edema (35.9%). Organ involvement was observed in 38 patients, with commonly affected organs being lymph nodes (78.9%), liver (94.7%), kidney (15.8%), heart (13.2%), and lung (7.9%). All patients had hematological abnormalities, primarily eosinophilia (69.2%) and atypical lymphocytosis (35.9%). Additional hematological changes included agranulocytosis (5.1%), hemophagocytic syndrome (5.1%), and pancytopenia (2.6%). Virus reactivation occurred in 21 patients (53.8%). The primary treatment for DRESS/DIHS due to SSZ is the immediate cessation of the drug, followed by systemic corticosteroid administration. Alternative treatments such as cyclosporine, intravenous immunoglobulin (IVIG), mycophenolate mofetil, cyclophosphamide, and rituximab require further investigation to establish their efficacy.

**Conclusion:**

SSZ may lead to DRESS/DIHS. To make a conclusive diagnosis, healthcare providers should conduct a thorough assessment by examining the patient’s clinical presentation, conducting physical evaluations, and analyzing laboratory findings. Immediate discontinuation of SSZ is recommended, and corticosteroids are often considered an efficacious treatment for DRESS/DIHS.

## Introduction

Sulfasalazine (SSZ), a drug with anti-inflammatory and immunosuppressive properties, undergoes breakdown in the ileocolonic tract to produce two main components: 5-aminosalicylic acid (5-ASA), the active therapeutic compound, and sulfapyridine, which serves as a carrier molecule (Azad Khan et al., 1977). SSZ is recommended for the treatment of autoimmune disorders such as ulcerative colitis (UC), Crohn’s disease (CD), and rheumatoid arthritis (RA) (Ye et al., 2024). The most frequently reported adverse drug reactions related to SSZ include nausea, vomiting, rash, headache, and abdominal pain, generally of mild intensity and well-tolerated. However, in recent years, it has been reported that SSZ may cause drug reactions with eosinophilia and systemic symptoms (DRESS) or drug-induced hypersensitivity syndrome (DIHS) (Shimada et al., 2023). SSZ-induced DRESS/DIHS is a severe adverse drug reaction characterized by multiorgan manifestations and reactivation of human herpesvirus-6 (Miyagawa and Asada, 2021). Clinical features of these syndromes encompass fever, rash, lymphadenopathy, internal organ involvement, and hematologic abnormalities such as lymphocytosis and eosinophilia (Yildirim Arslan et al., 2024). Despite the infrequent occurrence of DRESS/DIHS, its mortality rate can reach up to 10% (Mitsuno et al., 2024). Therefore, the early identification, diagnosis, and treatment of DRESS/DIHS are crucial to mitigate mortality rates. Of note, the understanding of SSZ-induced DRESS/DIHS is primarily based on case reports, which exhibit inconsistent and variable clinical features. Consequently, the diagnosis and treatment of SSZ-induced DRESS/DIHS present a significant challenge for healthcare professionals. The purpose of this study was to explore the characteristics of SSZ-induced DRESS/DIHS and provide insights for the diagnosis, treatment, and prognosis of SSZ-induced DRESS/DIHS.

## Methods

### Search strategy

We searched the literature related to SSZ-induced DRESS/DIHS by searching English databases (PubMed, Embase, Web of Science) and Chinese databases (Wanfang Data, China National Knowledge Infrastructure (CNKI)) from 1 January 2005, to 21 July 2024. The search keywords were “salazosulfapyridine” OR “sulfasalazine” OR “sulphasalazine” OR “SASP” OR “SSZ” AND “DRESS” OR “Drug reaction with eosinophilia and systemic symptoms” OR “Drug rash eosinophilia systemic symptoms” OR “DIHS” OR “Drug-induced hypersensitivity syndrome”.

### Inclusion and exclusion criteria

Inclusion criteria: case report and case series of SSZ-induced DRESS/DIHS. Exclusion criteria: reviews, mechanism studies, animal studies, duplicate cases, articles with insufficient data.

### Data extraction

Two researchers conducted an initial literature screening independently, adhering to predefined inclusion and exclusion criteria. Subsequently, a group discussion was held to finalize the selection of literature. Patient data extracted for analysis included region, gender, age, medical history, medication regimen, onset timing, clinical symptoms, laboratory tests, treatment modalities, and prognostic outcomes, facilitated through a self-designed data extraction table.

### Statistical analysis

The data were analyzed using descriptive statistics. Count data were presented as numbers and percentages, while measurement data were expressed as the median (minimum, maximum).

## Results

### Basic information

**FIGURE 1:**
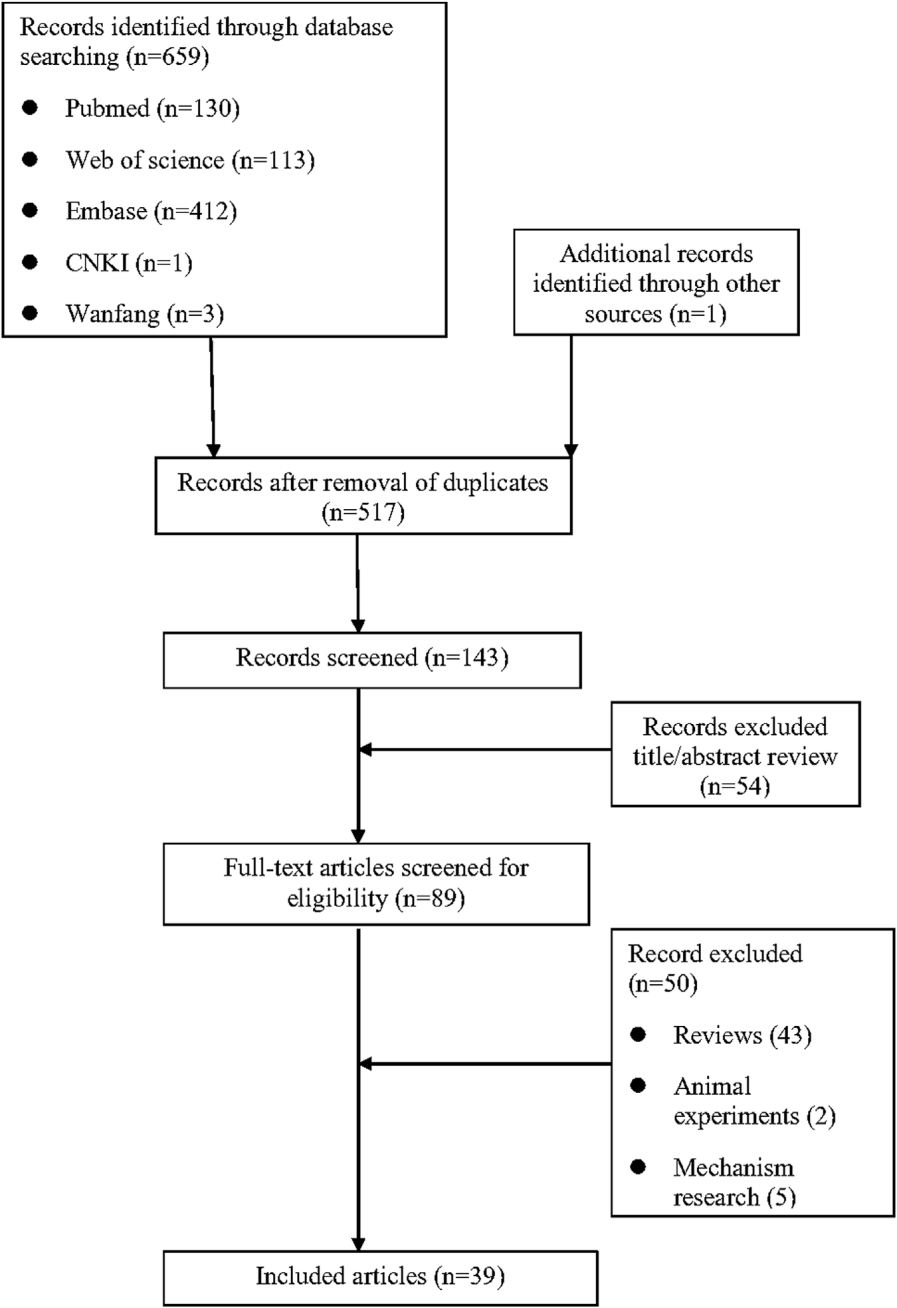
Flow diagram of the selection of studies for inclusion.

As shown in Figure 1, a total of 659 relevant studies were initially identified. After removing duplicate documents and screening the titles and abstracts, 39 studies were identified for a full-text assessment. The basic information of these 39 patients was summarized in Table 1 and the assessment of causality was carried out using the Naranjo scale (Naranjo et al., 1981). Furthermore, general data of patients was analyzed in Table 2. The study included 39 patients, comprising 15 males (38.5%) and 24 females (61.5%), with a median age of 47 years (range 11–82). The geographical distribution of these patients was as follows: 12 (30.8%) from Europe, 17 (43.6%) from Asia, 5 (12.8%) from America, 1 (2.6%) from Oceania, and 4 (10.2%) from Africa. The median onset time for DRESS/DIHS induced by sulfasalazine was 28 days (range 10–60). Among these patients, 30 cases (76.9%) were diagnosed with rheumatoid arthritis (RA), 7 cases (17.9%) with ulcerative colitis (UC), 1 case (2.6%) with ankylosing spondylitis (AS), and 1 case (2.6%) with Crohn’s disease (CD). Medical history data were available for 16 patients (41.0%), of whom seven had a history of infectious diseases and had taken antibiotics.

**TABLE 1:** The basic information of 39 patients reported in case series/reports.

References	Country or region	Gender/Age	Indications for SSZ	Daily dose (g)	Time of symptom onset(d)	Fever	Rash	Hematologic abnormalities	Other organ involvements	Viral reactivation	Treatment	Prognosis	Causality assessment
Descloux et al. (2005)	France	F/45	RA	NA	15	+	+	+	liver	-	steroids	recover	probable
Michel et al. (2005)	France	F/63	RA	NA	56	+	+	+	liver lymphadenopathy	HHV-6	steroids	recover	possible
Bejia et al. (2006)	Tunisia	F/46	RA	0.5	35	+	+	+	liver, lymphadenopathy	NA	steroids	recover	probable
Teo and Tan (2006)	India	F/49	RA	0.5	30	+	+	+	liver, lymphadenopathy	-	steroids	recover	probable
Furukawa et al. (2007)	Japan	F/43	RA	1	14	+	+	+	liver, lymphadenopathy	-	antihistamines, NSAIDs , steroids	recover	probable
Komatsuda et al. (2008)	Japan	F/58	RA	1	11	+	+	+	lymphadenopathy	EBV	steroids	recover	probable
Aquino et al. (2008)	Brazil	F/47	RA	0.5	56	+	+	+	liver, lymphadenopathy	-	steroids	recover	probable
Mennicke et al. (2009)	Switzerland	M/60	RA	NA	42	+	+	+	liver, kidney	HHV-6	cyclosporine, mycophenolate mofetil, steroids	death	possible
Augusto et al. (2009)	France	F/77	RA	1.5	28	+	+	+	lymphadenopathy, liver, kidney	HHV-6	steroids	recover	probable
Balci et al. (2009)	Turkey	M/15	RA	NA	28	+	+	+	lymphadenopathy, heart, liver	HHV-6	antihistamines, *N*-acetylcysteine, steroids	recover	probable
Rosenbaum et al. (2010)	Australia	F/11	UC	1.5	28	+	+	+	lymphadenopathy, liver	-	steroids	recover	probable
Bahat et al. (2010)	Turkey	F/68	RA	1	42	+	+	+	liver, kidney	-	steroids	recover	probable
Piñana et al. (2010)	Spain	M/11	RA	1	28	+	+	+	lymphadenopathy, liver, kidney	HHV-6	antihistamines, steroids	recover	possible
Miyazaki et al. (2011)	Japan	F/54	RA	1	12	+	+	+	lymphadenopathy, liver	HHV-6	steroids	recover	probable
Sekine et al. (2012)	Japan	F/56	RA	NA	28	+	+	+	-	HHV-6	steroids	recover	probable
Daoulah et al. (2012)	Saudi Arabia	F/56	AS	NA	21	+	+	+	lymphadenopathy, liver, heart	NA	cyclosporine, steroids	death	probable
Girelli et al. (2013)	Italy	F/53	RA	2	42	+	+	+	lymphadenopathy, liver	-	steroids	recover	possible
Hernández et al. (2013)	Spain	F/60	RA	2	21	+	+	+	liver, heart	EBV	steroids	recover	possible
Famularo et al. (2013)	Italy	M/45	RA	1	16	+	+	+	lymphadenopathy, liver	-	-	recover	probable
Phatharacharukul et al. (2013)	Thailand	M/24	RA	2	14	+	+	+	lymphadenopathy, liver	HHV-6	steroids	recover	probable
Fathallah et al. (2015)	Tunisia	M/25	UC	4	56	+	+	+	lymphadenopathy, liver	-	G-CSF, steroids	recover	probable
Kato et al. (2016)	Japan	F/82	RA	3	35	+	+	+	liver, heart	HHV-6	G-CSF, steroids	recover	probable
Sussman et al. (2017)	United States	M/16	UC	NA	28	+	+	+	lymphadenopathy, liver	NA	steroids	recover	possible
Ikari et al. (2017)	Japan	F/67	RA	0.5	30	+	+	+	lymphadenopathy, kidney	HHV-6; CMV	ganciclovir, steroids	recover	probable
Lang et al. (2017)	England	F/28	RA	2	14	+	+	+	lymphadenopathy, liver	HHV-7	paracetamol, steroids	recover	probable
Lian et al. (2018)	Singapore	M/59	RA	2	60	+	+	+	liver, kidney	CMV	cyclosporine, steroids	recover	probable
Swart et al. (2018)	South Africa	F/46	RA	NA	14	+	+	+	lymphadenopathy, liver, lung	NA	montelukast, promethazine, steroids	recover	probable
Morinaga et al. (2019)	Japan	M/67	RA	1	35	+	+	+	lymphadenopathy, liver, lung, heart	CMV; HHV-6	antihistamines, steroids	recover	possible
Itoi-Ochi et al. (2019)	Japan	M/61	RA	NA	14	+	+	+	lymphadenopathy, liver	HHV-6; CMV	steroids	recover	probable
Ammar et al. (2020)	Tunisia	M/45	UC	NA	45	+	+	+	liver, lymphadenopathy	HHV-6	steroids	recover	probable
Chiang et al. (2021)	Taiwan	F/47	RA	NA	30	+	+	+	lymphadenopathy, liver	HHV-6; CMV	cyclosporin, hydroxychloroquine, steroids	recover	probable
Winward et al. (2020)	United States	F/58	CD	NA	10	+	+	+	liver	-	-	recover	probable
Descamps et al. (2021)	France	M/32	RA	NA	21	+	+	+	lymphadenopathy, lung, liver	HHV-6	cidofovir, ganciclovir, immunoglobulins, steroids	recover	probable
Balconi et al. (2021)	Brazil	F/20	UC	NA	14	+	+	+	lymphadenopathy, liver	SARS-CoV-2	steroids	recover	probable
Sil et al. (2021)	India	M/30	RA	NA	60	+	+	+	lymphadenopathy, liver	-	antipyretic, antihistaminic, steroids, topical emollient	recover	probable
Hyser (2022)	United States	F/22	RA	NA	28	+	+	+	lymphadenopathy, liver	-	steroids	recover	possible
Chen et al. (2022)	China	M/52	UC	NA	21	+	+	+	lymphadenopathy, liver	NA	antihistamines, gamma globulin, steroids	recover	probable
Kanabaj et al. (2023)	Poland	M/42	UC	NA	28	+	+	+	lymphadenopathy, liver	-	antihistamines, steroids	recover	probable
Kuchinskaya et al. (2023)	Russia	F/12	RA	1.5	17	+	+	+	liver	HHV-6	immunoglobulin, steroids	recover	probable

+: positive; -: negative.

AS, ankylosing spondylitis; CD, Crohn’s disease; CMV, cytomegalovirus; EBV, epstein-barr virus; G-CSF, granulocyte colony-stimulating factor; HHV, human herpes virus; NA, not available; NSAIDs, non-steroidal anti-inflammatory drugs; RA, rheumatoid arthritis; SARS-CoV, severe acute respiratory syndrome coronavirus; UC, ulcerative colitis.

**TABLE 2:** General data of 39 patients reported in case series/reports.

Parameter		Value
Gender (39)^a^	Male	15 (38.5%)
	Female	24 (61.5%)
Age (39)^a^	Years	47 (11,82)
Region (39)^a^	Europe	12 (30.8%)
	America	5 (12.8%)
	Oceania	1 (2.6%)
	Asia	17 (43.6%)
	Africa	4 (10.2%)
Symptom onset time (39)^a^	days	28 (10,60)
Indication (39)^a^	RA	30 (76.9%)
	AS	1 (2.6%)
	UC	7 (17.9%)
	CD	1 (2.6%)
Disease history (16)^a^	Infectious diseases: urethritis, cholangitis, pharyngitis, hepatitis, pneumonia, lung tuberculosis, Lyme disease	7 (43.8%)
	Cardiovascular diseases: portal hypertension, hypertension, dyslipidemia, subarachnoid hemorrhage, cerebral infarction	3 (18.8%)
	Digestive system disease: cirrhosis, gastroesophageal reflux	4 (25%)
	Respiratory system disease: asthma, COPD	2 (12.5%)
	Reproductive system disease: ovarian cyst	1 (6.2%)
	Immune diseases: Sjögren syndrome, anterior uveitis, HIV, environmental allergies, Hashimoto disease	6 (37.5%)
	Metabolic diseases: type II diabetes	1 (6.2%)
Concomitant medications (23)^a^	Steroids: prednisone, prednisolone, methylprednisolone, deflazacort, budesonide, fluticasone furoate	14 (60.9%)
	Antibacterials: amoxicillin, amoxicillin clavulanate potassium, sulbactam-ampicillin, ceftriaxone, levofloxacin, ciprofloxacin, nalidixic acid, vancomycin, streptomycin, azithromycin, doxycycline, meropenem	7 (30.4%)
	Antitubercular agent: rifampicin, ethambutol	1 (4.3%)
	Analgesic: paracetamol, tramadol	1 (4.3%)
	NSAIDs: naproxen, diclofenac, celecoxib	5 (21.7%)
	Hypotensive drugs: valsartan and hydrochlorothiazide tablets	1 (4.3%)
	Hypoglycemic agent: metformin, repaglinide	1 (4.3%)
	PPI: omeprazole	2 (8.7%)
	Bronchodilators: salbutamol	1 (4.3%)
	Antipsychotics: risperidone	1 (4.3%)
	Antiepileptic drug: Levetiracetam	1 (4.3%)
	Anticoagulant: dabigatran	1 (4.3%)
	Anti-AIDS drug: tenofovir, lopinavir with ritonavir, lamivudine	1 (4.3%)
	Other drugs for RA: methotrexate, hydroxychloroquine	5 (21.7%)

^a^ Represents the number of patients out of 39 in whom information regarding this particular parameter was provided. AIDS, acquired immune deficiency syndrome; AS, ankylosing spondylitis; CD, Crohn’s Disease; COPD, chronic obstructive pulmonary disease; HIV, human immunodeficiency virus; NSAIDs, non-steroidal anti-inflammatory drugs; PPI, proton pump inhibitor; RA, rheumatoid arthritis; UC, ulcerative colitis.

### Clinical manifestations

The clinical symptoms of the 39 patients are detailed in Table 3. All these patients displayed fever and rash, with primary clinical manifestations including gastrointestinal symptoms in 15 cases (38.5%), comprising nausea, vomiting, diarrhea, anorexia, abdominal pain, and jaundice; and edema in 14 cases (35.9%), including facial and palate edema. Other symptoms consisted of autonomic system responses in 5 cases (12.8%), like asthenia, fatigue, and chills; severe skin involvement in 3 cases (7.7%), such as erythroderma, purpura, vitiligo, and alopecia; respiratory system responses in 3 cases (7.7%), like dyspnea and sore throat; neurological system responses in 2 cases (5.1%), such as syncope and headache; and myalgia in 4 cases (10.3%) and earache in 1 case (2.6%).

**TABLE 3:** Clinical information of 39 included patients.

Parameter	Clinical features	Value
Symptoms (39)^a^	Fever	39 (100%)
	Body temperature (25)^a^	39 (37.6, 41)^b^
	Rash	39 (100%)
	Digestive system: nausea, emesis, diarrhoea, anorexia, abdominal pain, Jaundice	15 (38.5%)
	Respiratory system: dyspnoea, sore throat	3 (7.7%)
	Neurological system: syncope, headache	2 (5.1%)
	Autonomic system: asthenia, fatigue, chill	5 (12.8%)
	Edema: facial edema, palate edema	14 (35.9%)
	Other skin lesions: erythroderma, purpura, vitiligo, alopecia	3 (7.7%)
	Other: myalgia, earache	5 (12.8%)
Other organ involvements (38)^a^	Lymphadenopathy	30 (78.9%)
	Liver	36 (94.7%)
	ALT (33)^a^ (U/L)	265 (20, 2471)^b^
	AST (30)^a^ (U/L)	198 (16, 4,258)^b^
	Aminotransferase group	
	Mild: (1.5–3.0) × ULN	4 (10.5%)
	Moderate: (3.0–5.0) × ULN	5 (13.2%)
	Serious: (5.0–20.0) × ULN	17 (44.7%)
	Life-threatening: >20.0 × ULN	5 (13.2%)
	Kidney	6 (15.8%)
	Lung	3 (7.9%)
	Heart	5 (13.2%)
Hematologic abnormalities (39)^a^	Leukocytosis	23 (59.0%)
	Neutrophilic leukocytosis	2 (5.1%)
	Lymphocytosis	12 (30.8%)
	Atypical lymphocytosis	14 (35.9%)
	Eosinophilia	27 (69.2%)
	Eosinophil count (×10^9^/L)	1.6 (0, 24.2)^b^
	Mononuclear leukocytosis	1 (2.6%)
	Agranulocytosis	2 (5.1%)
	Pancytopenia	1 (2.6%)
	Hemophagocytic syndrome	2 (5.1%)
Viral reactivation (21)^a^	HHV-6	16 (76.2%)
	HHV-7	1 (4.8%)
	CMV	5 (23.8%)
	EBV	2 (9.5%)
	SARS-CoV-2	1 (4.8%)

^a^ Represents the number of patients out of 39 in whom information regarding this particular parameter was provided.

^b^ Median (minimum-maximum).

ALT, alanine aminotransferase; AST, aspartate aminotransferase; CMV, cytomegalovirus; EBV, epstein-barr virus; HHV, human herpes virus; SARS-CoV, severe acute respiratory syndrome coronavirus; ULN, upper limit of normal.

### Physical examination and laboratory examination

The physical examination and laboratory examination of 39 DRESS/DIHS patients are summarized in Table 3. Physical examination revealed lymphadenopathy in 30 patients (78.9%) and laboratory tests revealed abnormal liver function in 36 patients (94.7%), including abnormal elevations of aminotransferase, bilirubin, or alkaline phosphatase. Six patients (15.8%) had abnormal renal function, including creatinine elevation, tubular dysfunction, or renal failure. Five patients (13.2%) had cardiac involvement, including pericardial effusion, tachycardia, heart failure, or acute ST-elevated myocardial infarction. And three patients (7.9%) developed lung disease, presenting with pneumonia or interstitial pneumonia. Furthermore, all 39 patients included in the study exhibited hematological abnormalities. Among them, 23 patients (59.0%) presented leukocytosis, 27 patients (69.2%) exhibited eosinophilia, 14 patients (35.9%) showed atypical lymphocytosis, 2 patients (5.1%) displayed neutrophilic leukocytosis, 1 patient (2.6%) manifested mononucleosis, 2 patients (5.1%) demonstrated agranulocytosis, and 2 (5.1%) patients developed hemophagocytic syndrome. Additionally, one patient (2.6%) presented with pancytopenia. Viral reactivations were experienced in 21 patients, including HHV-6 (16 cases, 76.2%), HHV-7 (1 case, 4.8%), CMV (5 cases, 23.8%), EBV (2 cases, 9.5%), and SARS-CoV-2 (1 case, 4.8%).

### Treatment and prognosis

The treatment and prognosis of the 39 DRESS/DIHS patients are summarized in Table 4. All patients promptly discontinued SSZ following the onset of symptoms. Furthermore, 37 patients (94.9%) were administered steroids, nine (23.1%) received antibiotics, seven (17.9%) were prescribed antihistamines, six (15.4%) were given immunosuppressants, three (7.7%) received immunoglobulins, and three (7.7%) underwent liver transplantation or hemodialysis or hemofiltration. Additionally, other drugs involved include antiviral drugs (2 cases, 5.1%), G-CSF (2 cases, 5.1%), NSAIDs (2 cases, 5.1%), PPI (1 case, 2.6%), paracetamol (1 case, 2.6%), glycyrrhizin (1 case, 2.6%), low molecular weight heparin (1 case, 2.6%), hydroxychloroquine (1 case, 2.6%), promethazine (1 case, 2.6%), montelukast (1 case, 2.6%), vitamin K (1 case, 2.6%), *N*-acetylcysteine (1 case, 2.6%), *clostridium* butyricum (1 case, 2.6%) and insulin (1 case, 2.6%). Ultimately, 37 patients (94.9%) achieved recovery while two (5.1%) died.

**TABLE 4:** Treatment and prognosis of 39 patients reported in case series/reports.

Parameter		Value
Treatment (39)^a^	Discontinued	39 (100%)
	Steroids	37 (94.9%)
	NSAIDs	2 (5.1%)
	Antihistamines	7 (17.9%)
	Immunosuppressor	6 (15.4%)
	Antibiotics	9 (23.1%)
	Antiviral agent	2 (5.1%)
	PPI	1 (2.6%)
	Paracetamol	1 (2.6%)
	G-CSF	2 (5.1%)
	Glycyrrhizin	1 (2.6%)
	Low molecular weight heparin	1 (2.6%)
	Hydroxychloroquine	1 (2.6%)
	Immunoglobulins	3 (7.7%)
	Promethazine	1 (2.6%)
	Montelukast	1 (2.6%)
	Vitamin K	1 (2.6%)
	*N*-acetylcysteine	1 (2.6%)
	*Clostridium* butyricum	1 (2.6%)
	Insulin	1 (2.6%)
	Other: liver transplantation, hemodialysis, plasma exchange	3 (7.7%)
Prognosis (39)^a^	Recover	37 (94.9%)
	Death	2 (5.1%)

^a^ Represents the number of patients out of 39 in whom information regarding this particular parameter was provided. G-CSF, granulocyte colony-stimulating factor; NSAIDs, non-steroidal anti-inflammatory drugs; PPI, proton pump inhibitor.

## Discussion

SSZ is a disease-modifying antirheumatic drug (DMARD) used for treating and managing autoimmune conditions such as rheumatoid arthritis and inflammatory bowel disease (Choi et al., 2024). It is also the second most common cause of DRESS/DIHS among drugs prescribed for rheumatic diseases (Adwan, 2017). In our analysis, the higher prevalence of SSZ-induced DRESS/DIHS in women compared to men may be attributed to gender-specific differences in the incidence of underlying conditions like rheumatoid arthritis. The onset of DRESS/DIHS is generally believed to occur between 2 and 8 weeks after starting the drug (Salame et al., 2019); however, we have observed cases with onset within 2 weeks (Komatsuda et al., 2008; Miyazaki et al., 2011; Winward et al., 2020). The reported mortality rate for DRESS/DIHS ranges from 3.8% to 10% (Chen et al., 2010; Hiransuthikul et al., 2016), which is consistent with our results (5.1%).

Initially associated with aromatic antiepileptic drugs, the DRESS/DIHS syndrome is now known to be induced by approximately 50 different drugs, including allopurinol, β-lactams, vancomycin, minocycline, fluoroquinolones, and sulfonamides (Cacoub et al., 2011; Wolfson et al., 2019). DRESS/DIHS is characterized by fever, rash, lymphadenopathy, elevated liver enzyme levels, and leukocytosis with eosinophilia. Previous studies have shown that 90% of DRESS/DIHS patients experience fever, although body temperature rarely exceeds 38.5°C (Ramirez et al., 2023). However, our analysis indicated that 100% of patients exhibited fever, with a median body temperature of 39°C. Additionally, 75% of patients presented with lymphadenopathy. The liver is the most commonly affected internal organ (75%), as evidenced by abnormal liver function tests. Kidney involvement was observed in 30% of patients, whereas lung involvement occurred in 25% (Calle et al., 2023; Kardaun et al., 2013). Similar to previous studies, our findings revealed that 78.9% of patients with DRESS/DIHS caused by SSZ exhibited lymph node involvement. However, in terms of internal organ involvement, our review showed that SSZ-induced DRESS/DIHS more frequently impacted the liver, with a prevalence of 94.7%. Liver function test results demonstrated median ALT and AST levels elevated by more than five times the normal limits, reaching 265 IU/L and 198 IU/L, respectively. In addition, we have clustered aminotransferase into groups according to the most recent version of the National Cancer Institute’s Common Terminology Criteria for Adverse Events (v5.0). According to our statistical analysis, among the cases in which specific data of aminotransferase were provided, abnormal elevation of aminotransferase was found in 31 patients, including 4 patients with mild liver damage, 5 patients with moderate liver damage, 17 patients with severe liver damage, and 5 patients with life-threatening condition. The risks of kidney, lung, and heart injuries were significantly lower. Previous reports indicate that the most common drugs causing kidney damage are allopurinol, carbamazepine, and dapsone. Minocycline is most frequently associated with lung injury, while heart involvement is more commonly linked to minocycline and ampicillin (Husain et al., 2013; Kardaun et al., 2013). Consequently, we suspect that different drugs induce DRESS/DIHS with varying manifestations of organ involvement, with SSZ more frequently affecting the liver. Furthermore, there are diverse hematological abnormalities than those previously reported in DRESS/DIHS. Kardaun et al. (2013) reported eosinophilia (95%) and atypical lymphocytes (67%) as the main characteristics of hematological abnormalities. However, our review indicated a prevalence of 69.2% for eosinophilia and 35.9% for atypical lymphocytes. Additionally, we discovered that 5.1% of patients had agranulocytosis, 5.1% had hemophagocytic syndrome, and 2.6% had pancytopenia. Although the proportion of patients with cytopenia is small, it warrants attention. Therefore, a complete blood cell count examination is indispensable when diagnosing DRESS/DIHS. Previous studies have demonstrated that herpes virus reactivation, especially HHV-6, is frequently depicted in DRESS/DIHS and has even been regarded as a criterion of DIHS by Japanese experts. It is considered to cause a more severe and/or prolonged course of the disease (Descamps et al., 2001; Kano et al., 2006; Picard et al., 2010; Shiohara et al., 2007; Shiohara et al., 2006; Suzuki et al., 1998; Tohyama et al., 2007). However, among the 39 cases we investigated, virus reactivation was involved in 21 cases. These included 16 cases of HHV-6 reactivation, 5 cases of CMV reactivation, 2 cases of EBV reactivation, 1 case of HHV-7 reactivation, and 1 case of SARS-CoV-2 reactivation. The rate of HHV-6 reactivation (41%) is lower than previously reported, particularly in Japan, where the probability exceeds 60% (Tohyama et al., 2007). This discrepancy may be due to incomplete information or the lack of examinations in some cases. Besides HHV-6 reactivation, we assert that the reactivation of viruses like CMV, EBV, or HHV-7 should also be considered at the time of diagnosis.

In the treatment of DRESS/DIHS resulting from SSZ, the immediate cessation of SSZ is paramount. Following this, systemic administration of corticosteroids is essential (Brüggen et al., 2024). It is recommended to start with a dose of at least 1 mg/kg/day of prednisone or its equivalent, and tapering should be implemented over 3–6 months (Gottlieb et al., 2022). If this regimen proves insufficient, intravenous pulse methylprednisolone may be considered (Ramirez et al., 2023). In a case presented by Augusto et al. (2009), the patient discontinued corticosteroids after 20 days, leading to secondary renal failure. Therefore, rapid dose reduction of corticosteroids is not advised to prevent disease recurrence. In fact, the latest international consensus recommends that mild and moderate DRESS should have steroids reduced gradually over a period of 6 weeks to 3 months; severe DRESS should have steroids reduced gradually over a period of 3–6 months (Brüggen et al., 2024). Regular follow-up visits after discharge are also crucial. Additionally, case reports suggest alternative treatments including cyclosporine, intravenous immunoglobulin (IVIG), mycophenolate mofetil, cyclophosphamide, and rituximab (Stirton et al., 2022). However, further research is needed to establish the efficacy of these alternative medications.

## Conclusion

DRESS/DIHS is a rare but serious adverse effect of SSZ. Patients receiving SSZ treatment must consistently monitor their symptoms. If adverse reactions such as unexplained rash, fever, abdominal pain, jaundice, facial edema, or lymph node enlargement occur, they should immediately seek medical attention for timely intervention and early treatment, thereby minimizing the detrimental effects of SSZ-induced DRESS/DIHS. Clinicians should promptly diagnose DRESS/DIHS when these symptoms arise and immediately discontinue SSZ use, employing corticosteroids for treatment. A complete blood cell count, examinations of the liver, kidney, heart, and lungs, as well as tests for viral reactivation should be performed.

## Data Availability

The original contributions presented in the study are included in the article/supplementary material, further inquiries can be directed to the corresponding authors.
